# Clinicopathological Significance of CD44 and CD117 Expression/Co-Expression in Invasive Ductal Breast Cancer

**DOI:** 10.3390/diagnostics16203401

**Published:** 2026-10-19

**Authors:** Miodrag Djordjevic, Aleksandar Karanikolic, Ljubinka Velickovic

**Affiliations:** 1 Faculty of Medicine, University of Niš, 18000 Nis, Serbia; aleksandarkaranikolic@gmail.com (A.K.); ljubinkavelickovic60@gmail.com (L.V.); 2 Clinic of Endocrine Surgery, University Clinical Center, Bul. Dr Zorana Djindjica 48, 18000 Nis, Serbia; 3 Center for Pathology, University Clinical Center, 18000 Nis, Serbia

**Keywords:** CD44, CD117, axillary node status, breast cancer

## Abstract

**Background/Objectives**: Breast cancer (BC) is the most common cancer in women worldwide and has a high mortality rate; thus, it is essential to determine the relevance of not only standard clinicopathological parameters, but also non-standard parameters, such as cancer stem cell molecular markers CD44 and CD117. These factors could possibly be used to predict cancer aggressiveness and metastasis. The present study aimed to evaluate the significance of CD44 and CD117 expression/co-expression in relation to clinicopathological parameters, molecular cancer subtypes, and axillary lymph node (ALN) status in invasive ductal cancer (IDC) of the breast. **Methods**: The investigation included 136 patients with IDC in whom radical mastectomy had been performed. The level of CD44 and CD117 expression in cancer tissue (high or low) was determined via immunohistochemical analysis. **Results**: High CD117 expression and CD44/CD117 co-expression were closely linked to a higher Ki-67 index (*p* < 0.001; *p* = 0.003, respectively) and triple-negative breast cancer (TNBC) (*p* < 0.001; *p* < 0.001, respectively). Multivariate linear regression analysis revealed that CD44/CD117 co-expression was independently associated with a higher number (*p* < 0.001), as well as a higher percentage (*p* = 0.005) of metastatic ALNs. **Conclusions**: This study showed that CD44/CD117 co-expression is an independent predictor of a higher number and percentage of metastatic ALNs in IDC and may have potential prognostic value.

## 1. Introduction

Breast cancer (BC) is the most common malignancy among women worldwide and a leading cause of cancer-related mortality [1]. It is a heterogeneous disease caused by various histological types with different molecular characteristics. Intratumoral heterogeneity arises from genomic, epigenomic, transcriptomic, proteomic, and microenvironmental alterations [2], affecting key tumor properties such as proliferation, apoptosis, metastasis, and response to therapy [3]. Studies on human carcinogenesis have shown that a small subpopulation of cancer stem cells (CSCs), constituting 0.1–1% of the cancer mass, contributes to intra-tumoral heterogeneity and the modulation of its biological profile. CSCs across different tumors are characterized by the capacity for self-renewal and the ability to differentiate into a predominant cancer non-stem cell population [4]. Overexpression of transcription factors is responsible for the transition of a cell’s state from somatic to induced pluripotent stem (iPS) cells; this means that non-CSCs can acquire stem-like properties through de-differentiation or reprogramming processes [5,6].

The major signaling pathways regulating breast cancer stem cell (BCSC) characteristics are Wnt, PI3K/Akt/m TOR, Notch, Hedgehog, and HER2 [4]. Genetic mutations in encoding proteins involved in these pathways in normal stem cells are responsible for the transformation of these cells into BCSCs and for subsequent uncontrolled cell proliferation and tumor progression [7]. Accumulating evidence has shown that BCSCs contribute to the progression of the disease and poor therapeutic response. They may represent the residual cell population responsible for the development of secondary (recurrent or metastatic) tumors. CD44 and CD117 proteins are stemness-related surface markers of BCSCs.

CD44, a non-kinase transmembrane glycoprotein, is expressed by normal stem cells, cancer stem cells and cancers. Hyaluronic acid (HA), the major extracellular matrix component, is the most specific ligand for CD44, and the HA-CD44 complex activates signaling pathways, such as Ras-MAPK and PI3K/Akt, that are involved in tumor proliferation and progression. In addition, CD44 acts as a co-receptor of various growth factors (i.e., HGF and VEGFG-2) [8,9].

Proto-oncogene c-kit (CD117) is a transmembrane tyrosine kinase receptor and can be activated through binding to a ligand called stem cell factor (SCF). Activated CD117 plays an important role in regulating cellular activities, such as cell differentiation, proliferation, survival, adhesion, motility, and angiogenesis. CD117 exhibits oncogenic potential, achieved through overactivation or constitutive mutations, mostly in the tyrosine kinase domain [10].

Invasive ductal cancer (IDC) of the breast is the most prevalent histological subtype [11], which exhibits considerable variability in its clinical presentation. Based on immunohistochemistry, BCs are divided into the following subtypes according to the presence of estrogen receptor (ER), progesterone receptor (PR), and human epidermal growth factor receptor 2 (HER2): luminal A, luminal B, HER2- positive, and triple-negative breast cancer (TNBC) [12]. BCSCs are not equally represented across all molecular subtypes of IDC. The highest proportion of BCSCs is found in luminal B, followed by TNBC and the HER2- positive subtype, while the lowest proportion of BCSCs is observed in the luminal A subtype [12], which is characterized by lower aggressiveness and a more favorable prognosis. Different cancer subtypes feature distinct BCSC markers. CD44, as the most commonly investigated BCSC marker, has been reported to be the most prevalent in the basal-like subtype, followed by HER2-positive, non-basal TNBC, luminal B, and luminal A subtypes [13]. CD117 is also associated with TNBC and the basal-like subtype [14,15]. However, the prognostic value of BCSC markers is still under investigation. The available evidence concerning the association of CD44 and CD117 expression with clinicopathological parameters and axillary lymph node (ALN) metastasis in breast cancer remains inconclusive, with studies reporting conflicting findings. In addition, previous studies have predominantly focused on the expression of the CD44 marker on its own or in combination with CD24 and ALDH1, whereas CD117 has been evaluated separately as a prognostic marker in breast cancer. Thus, data regarding CD44/CD117 co-expression in invasive ductal cancer remain scarce.

Therefore, this study aimed to evaluate the clinical relevance of CD44 and CD117 expression and co-expression in IDC of the breast by analyzing their correlation with clinicopathological parameters and molecular subtypes, as well as their association with ALN status.

## 2. Materials and Methods

### 2.1. Study Population

The study population included 136 consecutive female patients with primary IDC of the breast, aged on average 64.01 ± 11.92 years (mean ± SD), in whom radical mastectomy had been performed at the Clinic of Endocrine Surgery, University Clinical Center (UCC) Nis. The included patients did not have multicentric or multifocal tumors or distant metastases, and they did not receive any preoperative neoadjuvant oncological therapy. The luminal B subtype was excluded from analysis due to the limited number of cases, which could have led to unreliable and statistically unstable results.

### 2.2. Histopathological Analysis

Histopathological analysis of biopsy samples (breast tissue and axillary contents) was used to evaluate the following: histological type and molecular subtype of cancer, the presence of ALN metastases (yes or no), pN stage, grade (G), Nottingham score, ER, PR and HER2 status, and Ki-67 proliferation index.

The axillary lymph node (pN) stages were defined as follows: pN0 (absence of metastases); pN1 (metastases in 1–3 ALNs); and pN2,3 (metastases in 4 or more ALNs). In the group of patients with metastases, the average number and percentage of positive ALNs were calculated. The percentage of positive ALNs was obtained as the ratio of metastatic ALNs to the total number of examined lymph nodes.

### 2.3. Immunohistochemical Analysis

**Figure 1:**
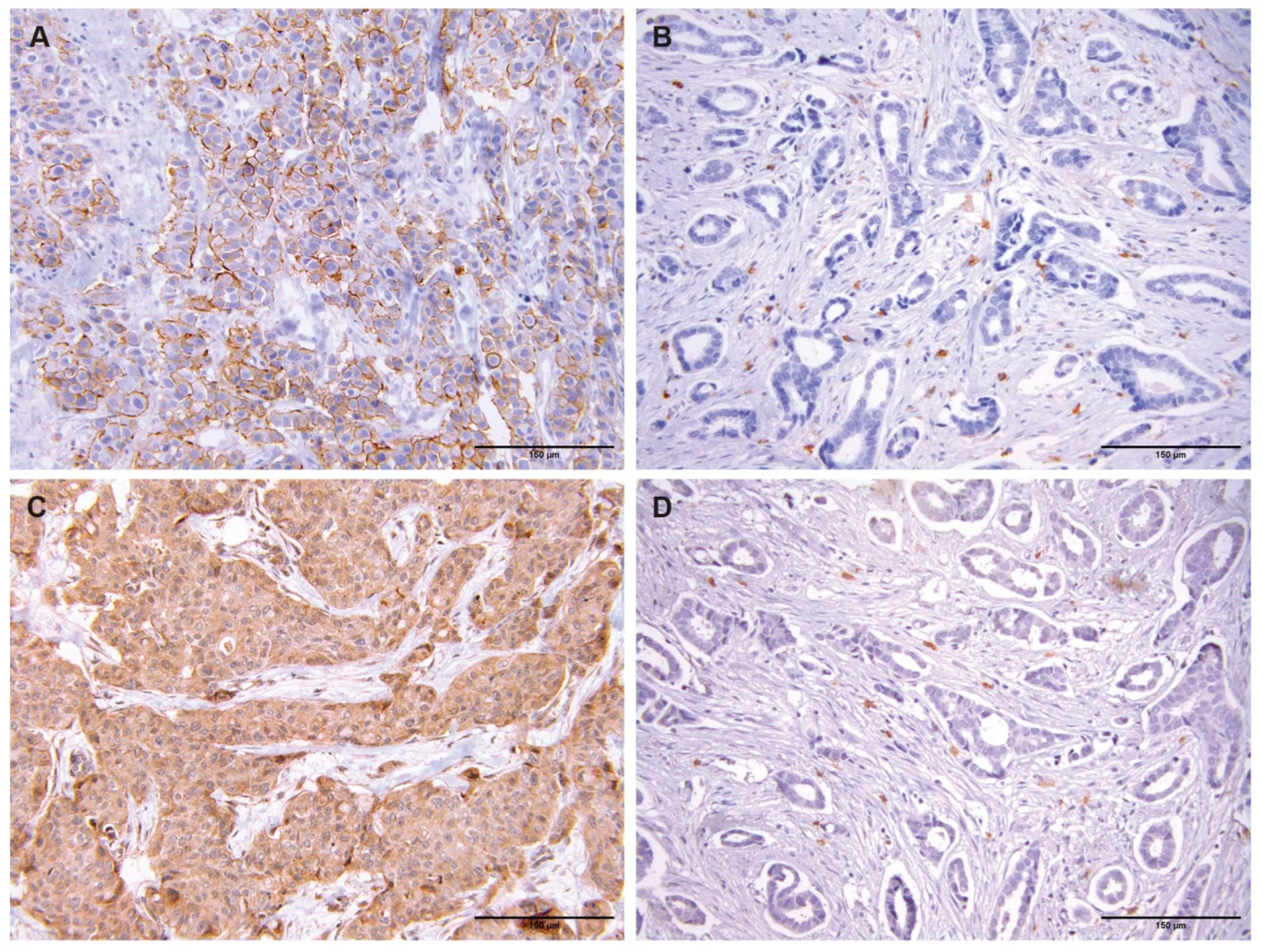
Representative immunohistochemical staining of CD44 and CD117 in invasive ductal breast cancer. High expression was defined as intermediate or strong staining intensity, whereas low expression was defined as negative or weak staining intensity. (**A**) High CD44 expression; (**B**) low CD44 expression; (**C**) high CD117 expression; (**D**) low CD117 expression. Original magnification: ×200. Bar 150 µm (**A**–**D**).

Breast cancer tissue specimens were obtained from the Center for Pathology, UCC Nis, for immunohistochemical analysis of molecular marker expression. Immunohistochemical examination of molecular marker expression, based on whole slides, was performed on 4 µm thick sections prepared from representative formalin-fixed, paraffin-embedded tumor blocks for each patient. All samples were reviewed by a pathologist to ensure the presence of representative tumor areas. Tissue sections were deparaffinized, rehydrated, heat-pretreated using retrieval solution, and incubated with primary polyclonal rabbit Anti-Human CD117 antibodies, c-kit (catalog number A4502, Dako, Glostrup, Denmark, dilution 1:200) and monoclonal mouse Anti-Human CD44 antibodies (catalog number M7082, clone DF1485, Dako, Denmark, 1:50 dilution). After washing, appropriate secondary antibodies were applied, followed by visualization using diaminobenzidine-tetrachloride and counterstaining by section staining with Mayer’s hematoxylin. Negative controls were included in all immunohistochemical procedures by substituting the primary antibody with PBS. In addition, certain normal tissue structures served as internal positive controls. Brownish membranous staining represented a positive finding. For each of the analyzed markers, the percentage of cells with a positive reaction was determined in relation to the total number of tumor cells per section. Brown pigment staining intensity was scored from 0 to 3 in the following way: 0—absence of reaction; 1—weak; 2—moderate; and 3—strong staining. The final score of immunohistochemical analysis of molecular marker expression was evaluated semi-quantitatively based on the percentage of positively stained tumor cells and the intensity of the staining under low and high power magnification (200× and 400×). CD117 expression was considered high if the tumor demonstrated marker expression in more than 10% of tumor cells with intermediate or strong staining [16]. CD44 expression was considered high if the tumor demonstrated expression in 50% or more cancer cells with intermediate or strong staining [17,18] (Figure 1). Histopathological slide review, immunohistochemical evaluation, and scoring of all cases were performed independently by an experienced pathologist who was blinded to the clinicopathological data.

### 2.4. Statistical Analysis

All statistical analyses were performed using SPSS version 18.0 (IBM Corp., Armonk, NY, USA).

The chi-square test of independence was used to compare the frequency of categories of attributive characteristics between the groups. The statistical assumptions for the chi-square analysis were evaluated based on minimum expected cell frequencies. When the assumption was violated due to sparse data (i.e., any expected cell frequency fell below 5), Fisher’s exact test was used instead. All categorical clinicopathological variables, including tumor size (T1–T4) and axillary lymph node pN stages (pN0, pN1, pN2, pN3), were evaluated using their original standardized classifications without sub-category grouping or merging, thereby avoiding cell-collapsing bias. For variables with more than two categories showing a statistically significant overall *p*-value, post hoc evaluation was performed through direct cell-by-cell inspection of observed versus expected frequencies within the contingency matrix to identify the specific categorical differences.

Continuous variables (Ki-67 index, number of metastatic ALNs, percentage of metastatic ALNs, Nottingham score, ER score, and PR score) were tested for normality using the Shapiro–Wilk test. Since all deviated from normality, group comparisons were performed using the Mann–Whitney U test (for two groups).

All tests were two-sided, and *p*-values < 0.05 were considered statistically significant. No adjustment for multiple comparisons was applied to the primary exploratory analyses, and *p*-values between 0.01 and 0.05 were considered exploratory.

To assess the potential impact of multiple testing, a sensitivity analysis using the Bonferroni correction was additionally performed for prespecified families of parallel bivariate comparisons. Specifically, four families were defined: one comprising the three comparisons of CD44 expression, CD117 expression, and the number of highly expressed markers with ALN status in Table 1, and three comprising the 12 clinicopathological comparisons with CD44 expression, CD117 expression, and CD44/CD117 co-expression in Table 2. For each family, Bonferroni-adjusted *p*-values were calculated by multiplying the original *p*-value by the number of tests within that family, with adjusted values >1 set to 1.00. The adjusted results were used as a sensitivity analysis to evaluate the robustness of the observed associations and were not used to redefine the primary exploratory analyses.

Univariate and multivariate logistic regression analyses were performed to examine the association between individual factors and the probability of ALN involvement. Odds ratios (ORs) and their corresponding 95% confidence intervals (CIs 95%) for the presence versus absence of ALN involvement were calculated.

Univariate and multivariate linear regression analyses were used to examine the association between individual factors and the number and percentage of metastatic ALNs. Linear correlation (B) coefficients and their 95% confidence interval limits (CIs 95%) were calculated. Because the number of metastatic ALNs and the percentage of involved nodes were treated as continuous variables, linear regression was performed to analyze their associations with individual factors. For multivariable logistic and linear regression models, tumor size, histological grade, Ki-67 index, molecular subtype, CD44 expression, CD117 expression, and their co-expression were initially entered as candidates. To avoid substantial redundancy and potential collinearity among predictors, ER, PR, and HER2 status were not included in the initial multivariate models since molecular subtype is defined on the basis of those variables. Backward elimination using the Wald method was applied, with a removal threshold of *p* > 0.10. Only variables with *p* < 0.05 were retained in the final model after backward elimination. We also checked for multicollinearity (VIF < 2.5 for all retained variables) and assessed model fit (Hosmer–Lemeshow test for logistic regression; adjusted R^2^ for linear regression). Residuals for linear regression models were approximately normal.

Because the number of metastatic ALNs represents overdispersed count data, negative binomial regression analysis was performed as a sensitivity analysis. The first model evaluated the absolute number of metastatic ALNs, with the number of metastatic ALNs entered as the dependent variable. The second model evaluated the rate of metastatic ALNs relative to the total number of examined ALNs. In this model, the number of metastatic ALNs was entered as the dependent variable, and the logarithm of the total number of examined ALNs per patient was included as an offset variable. Thus, the model estimated the rate of metastatic ALNs per examined lymph node while accounting for differences in the total number of examined nodes between patients. Both models included tumor size, histological grade, molecular subtype, Ki-67 index, and CD44/CD117 co-expression as independent variables. Negative binomial regression results are presented as incidence rate ratios (IRRs) with corresponding 95% confidence intervals.

The primary rationale for employing multiple linear regression models for the number and percentage of metastatic ALNs was based on the standard post-estimation diagnostics: the regression residuals for both outcome models demonstrated approximate normality, and the variance inflation factor (VIF) indicated no evidence of multicollinearity (VIF < 2.5 for all variables). However, because the absolute number of involved lymph nodes represents discrete count data susceptible to overdispersion, and the percentage represents bounded data, linear models can sometimes under-represent variance. To address these potential limitations and assess the robustness of our conclusions, negative binomial regression analyses were conducted as a sensitivity analysis. The primary linear models and the secondary sensitivity models share a complementary relationship: the linear regression quantifies the absolute linear shift (B coefficients), whereas the negative binomial regression models the rate changes (Incidence Rate Ratios) while controlling for overdispersion. The negative binomial analyses were performed as a sensitivity analysis to assess whether the main associations remained consistent under an alternative model appropriate for count and rate data.

For categorical variables, the designated reference category was used as the comparator for all other categories in all the regression models.

## 3. Results

ALN metastases were detected in 77 (56.6%) out of 136 patients. Axillary lymph node evaluation revealed pN0, pN1, and pN2/N3 stages in 59 (43.4%), 62 (45. 6%), and 15 (11.0%) patients, respectively.

Analysis of CD44 and CD117 expression, as well as the number of markers with high expression (one or both) in relation to ALN status, failed to uncover a significant association between high expression of these markers or between the number of markers and ALN status (Table 1).

The correlations between clinicopathological cancer characteristics and the expression of CD44 and CD117, as well as their co-expression, are shown in Table 2.

Cancers with high CD44 had a significantly higher mean Ki-67 index (33.23 ± 17.84 vs.26.53 ± 13.73, *p* = 0.020), whereas the mean ER (2.68 ± 3.71 vs. 4.28 ± 3.76, *p* = 0.024) and PR scores (2.28 ± 3.23 vs. 3.92 ± 3.52, *p* = 0.012) were significantly lower. Regarding the molecular subtype of IDC, in patients with high CD44, the prevalence of TNBC was significantly higher (45.0 vs. 18.8%), whereas the prevalence of the luminal A subtype was significantly lower (35.0 vs. 57.3%) compared with those with low CD44 expression (*p* = 0.006).

Cancers with high CD117 also had a significantly higher mean Ki-67 index (37.17 ± 17.90 vs. 26.15 ± 13.69, *p* < 0.001) and lower mean ER (1.59 ± 3.17 vs. 4.41 ± 3.75, *p* < 0.001) and PR scores (1.31 ± 2.66 vs. 4.01 ± 3.50, *p* < 0.001) compared to cancers with low CD117 expression. High CD117 was significantly associated with TNBC (65.5 vs. 15.9%), whereas low CD117 expression was significantly associated with the luminal A subtype (58.9 vs.20.7%) (*p* < 0.001).

In patients with high expression of both markers (CD44/CD117 co-expression), a significantly higher number of metastatic ALNs (5.33 ± 7.06 vs. 1.85 ± 2.66, *p* = 0.001) and percentage of involved nodes (31.37 ± 40.54 vs. 14.20 ± 19.46, *p* = 0.011) were detected compared to those with low expression of these proteins or those with only one highly expressed protein (CD44 or CD117). CD44/CD117 co-expression was significantly associated with lower mean ER (0.58 ± 2.02 vs. 4.12 ± 3.80, *p* = 0.002) and PR scores (0.42 ± 1.44 vs. 3.73 ± 3.52, *p* = 0.002), a higher mean Ki-67 index (41.00 ± 18.12 vs. 27.29 ± 14.52, *p* = 0.003), a higher prevalence of TNBC (83.3 vs. 21.0%), and a lower prevalence of the luminal A subtype (8.3 vs. 54.8%) (*p* < 0.001).

Sensitivity analysis using the Bonferroni procedure showed that none of the associations between CD44 expression and the clinicopathological variables remained statistically significant after adjustment. In contrast, the associations between CD117 expression and ER (adjusted *p* = 0.004) and PR scores (*p* = 0.002), Ki-67 index (*p* = 0.006), and molecular subtype (*p* < 0.001) remained statistically significant. For CD44/CD117 co-expression, the associations with the number of involved ALNs (*p* = 0.012), ER (*p* = 0.024) and PR scores (*p* = 0.024), Ki-67 index (*p* = 0.036), and molecular subtype (*p* < 0.001) remained statistically significant, whereas the association with the percentage of metastatic ALNs did not remain significant after adjustment (adjusted *p* = 0.132).

Univariate logistic regression analysis demonstrated that an increase probability of ALN metastasis was associated with stage T4 (OR = 7.200; 95% CI: 1.850–28.016; *p* = 0.004) and a higher mean Ki-67 index (OR = 1.022; 95% CI: 1.001–1.044; *p* = 0.049), but not with high CD44 and CD117 expression or their co-expression. Multivariate logistic regression analysis identified the T4 tumor stage as a significant predictor of ALN metastasis development (Table 3).

Univariate linear regression analysis revealed that a higher number of involved ALNs was significantly correlated with lower ER (B = −0.151; 95% CI: −0.302 −0.001; *p* = 0.048) and PR scores (B = −0.189; 95% CI: −0.351 −0.027; *p* = 0.022), as well as with CD44/CD117 co-expression (B = 3.478; 95% CI: 1.532–5.425; *p* = 0.001). Multivariate linear regression analysis identified the T4 tumor stage (B = 1.671; 95% CI: 0.162–3.181; *p* = 0.030) and CD44/CD117 co-expression (B = 3.609; 95% CI: 1.686–5.532; *p* < 0.001) as significant predictors of a higher number of metastatic ALNs. The VIF values were 1.004 for both T4 and CD44/CD117 co-expression (Table 4).

Additional negative binomial regression analysis confirmed that CD44/CD117 co-expression was independently associated with a higher number of metastatic ALNs (IRR = 3.289; 95% CI: 1.567–6.897; *p* = 0.002) after adjustment for tumor size, molecular subtype, histological grade, and Ki-67 index. Tumor size (T3-T4) (IRR = 2.770; 95% CI: 1.437–5.348; *p* = 0.002) and Ki-67 index (IRR = 1.030; 95% CI: 1.007–1.054; *p* = 0.011) were also independently associated with a higher number of metastatic ALNs. The luminal A subtype was associated with a lower number of metastatic ALNs (IRR = 0.279; 95% CI: 0.117–0.666; *p* = 0.004) (Table 5).

Univariate linear regression analysis demonstrated that a higher percentage of metastatic ALNs was significantly associated with the T4 tumor stage (B = 15.923; 95% CI: 5.715–26.132; *p* = 0.002), reduced ER (B = −1.066; 95% CI: −2.057 −0.075; *p* = 0.035) and PR scores (B = −1.257; 95% CI: −2.327 −0.187; *p* = 0.022), and CD44/CD117 co-expression (B = 17.170; 95% CI: 4.034–30.306; *p* = 0.011). Multivariate linear regression analysis identified the T4 tumor stage (B = 16.811; 95% CI: 6.850–26.772; *p* = 0.001) and CD44/CD117 co-expression (B = 18.481; 95% CI: 5.791–31.170; *p* = 0.005) as significant predictors of a higher percentage of metastatic ALNs. The VIF values were 1.004 for both T4 and CD44/CD117 co-expression (Table 6).

Negative binomial regression analysis with a log offset showed that CD44/CD117 co-expression (IRR = 2.457;95% CI: 1.629–3.717; *p* < 0.001) and a higher Ki-67 index (IRR = 1.040; 95% CI: 1.022–1.058; *p* < 0.001) were independently associated with a higher rate of metastatic ALNs. Tumor size, histological grade, and molecular subtype were not significantly associated with the rate of metastatic ALNs (Table 7).

## 4. Discussion

CD44 has been extensively investigated as a marker in BC patients, with some authors reporting that CD44 overexpression is associated with ALN metastases [19]. Vadhan et al. identified that metastatic ALNs exhibit higher expression of the CD44 protein compared to primary tumors and that migration and invasion abilities were increased in CD44-overexpressing cells [20]. However, some authors have asserted that there is no significant association between CD44 overexpression and the development of metastases in BC. Punhani et al. demonstrated that CD44 overexpression is only significantly correlated with the histological grade of TNBC [21], while Jang et al. found that CD44 expression was not associated with clinicopathological parameters including histological grade, tumor size and stage, and ALN metastases [22]. Recent data reported by Roosta et al. also revealed no significant association between CD44 expression and any clinicopathological parameters, except for BC stage [23].

In this study, high CD44 was detected in 29.41% of patients and low in 70.58% of patients. Associations between high CD44 and locoregional cancer spread, pN stage, and the number and percentage of involved ALNs could not be established. In unadjusted analyses, high CD44 expression was associated with a higher Ki-67 index (*p* = 0.020), lower mean ER (*p* = 0.024) and PR scores (*p* = 0.012), and the TNBC subtype of IDC (*p* = 0.006). However, none of these associations remained statistically significant after Bonferroni correction. Therefore, these findings should be considered exploratory and hypothesis-generating. Nevertheless, the observed association with TNBC is consistent with previous reports, including that of Tomar et al., who demonstrated that CD44 expression is significantly more prevalent in TNBC than in other subtypes of IDC (75% vs. 53%) [24]. The association between CD44 expression and TNBC subtype may be attributed to the high BCSC content in TNBC and the basal-like subtype, characterized by CD44 as the most common marker [4,7].

Some studies demonstrated that CD44 is associated with metastasis and poor prognosis in TNBC [7,19]. One meta-analysis demonstrated that increased CD44 expression (measured through mRNA and protein levels) in the basal-like subtype is correlated with the epithelial–mesenchymal transformation and CSC gene profile [25]. Abnormal Notch signaling is also associated with TNBC, and increased tumor aggressiveness is correlated with Notch receptor overexpression [26].

The CD117 protein is present in the normal breast epithelium, in proliferative lesions, and in cancer. CD117 is reduced upon malignant transformation of the breast epithelium, and it is thought that CD117 has a role in breast carcinogenesis [27].

In a study by Amin et al., CD117 overexpression was detected in about 29% of invasive breast cancers. Absence of CD117 was associated with aggressive breast tumor characteristics, such as increased blood vessel density, large tumor sizes, higher histological grade, a higher number of metastatic lymph nodes, and ER and PR negativity [28]. Tsutsui et al. also found a significant correlation between the lack of CD117 expression and metastases in lymph nodes [29], and Raikar et al. showed a significant association between negative CD117 expression and higher tumor grades in IDC [30]. In contrast to the above, Simon et al. reported a significant association between CD117 overexpression and high histological grades, but not with tumor size, ALN pN stage, or patient survival [31]. The results of a recent study showed no correlation between CD117 expression and clinicopathological parameters (tumor size, grade, and pN status) [32].

In this study, in most patients, tumors demonstrated low CD117 expression (78.7%), which supports the findings of Amin et al. High expression of CD117 was detected in 21.3% of patients, but significant associations between high/low CD117 expression and the development of ALN metastases, pN stage, and the number and percentage of involved ALNs could not be established. High CD117 expression was significantly correlated with TNBC, a higher Ki-67 index, and low ER and PR scores. Our results concerning the correlations of CD117 overexpression with TNBC are in agreement with those of other studies [14,33].

The contradictory findings regarding the correlation of CD44 and CD117 with clinicopathological parameters and their impact on ALN status may be attributed to the heterogeneity in tumor molecular subtypes, variable marker detection methodologies, and the lack of standardized quantification across different studies. A standardized protocol for the routine clinical detection of cancer stem cell markers should be developed in the future.

The results presented in the literature suggest an important role of molecular marker co-expression in cancer progression. Pagotto et al. indicated that ovarian cells with CD44/CD117 co-expression had greater potential to express stem-associated transcription factors such as NANOG, SOX2, and OCT4 compared to CD44^+^/CD117^−^ cells [34].

The results of this study indicate that high expression of both membranous markers is significantly associated with a higher number of metastatic ALNs, a higher percentage of involved ALNs, and a higher Ki-67 index, but with lower ER and PR scores. High expression of both membranous markers was also significantly correlated with TNBC. However, the association with the percentage of metastatic ALNs did not remain significant after Bonferroni correction.

Univariate linear regression analysis showed that CD44/CD117 co-expression was significantly associated with a higher number (*p* = 0.001) and percentage of involved ALNs (*p* = 0.011), while multivariate linear regression analysis, which excluded ER, PR, and HER2 status due to substantial redundancy or collinearity with molecular subtype, demonstrated that CD44/CD117 co-expression is independently associated with a higher number and percentage of metastatic ALNs. Negative binomial regression models confirmed significant associations between CD44/CD117 co-expression and the number and rate of metastatic ALNs, suggesting its potential role as a marker of more aggressive disease. However, the contribution of uniformly expressed markers remains an important determinant of metastatic potential. Further studies are needed to elucidate their correlation with stemness-related markers.

An important and interesting result of this study is the absence of an association between CD44/CD117 co-expression and the presence of metastases, contrasted with its significant correlation with the number and percentage of metastatic ALNs. It can be hypothesized that these findings likely reflect distinct biological mechanisms underlying early dissemination and metastatic outgrowth. Increasing evidence suggests that the initial steps of metastasis are determined by dynamic processes such as the epithelial–mesenchymal transition (EMT) and reciprocal interactions with the tumor microenvironment (TMA) and the immune system. [35,36,37]. The EMT represents a typical process of cellular plasticity, characterized by a reversible ability of epithelial cell dedifferentiation or transdifferentiation into mesenchymal cells, followed by morphodynamical and phenotypic changes [35].

EMT plasticity generates different phenotypes (defined by the degree of epithelial and mesenchymal cell markers) [35]. However, EMT, as a dynamic process, enables cancer cells to shift between various phenotypes (M, E and hybrid E/M phenotypes), with the possibility that they may remain in a stable phenotypic state.

CSCs from the primary tumor transform into mesenchymal-like circulating tumor cells (CTCs) through EMT and then enter the circulation [38]. Through the conversion of epithelial cells, as non-mobile cells, into mesenchymal cells, EMT facilitates invasion, migration, and intravasation into circulation [39]. In CTCs, CD44 has a role in cell–cell interactions, forming clusters that facilitate cell migration [40]. Once at the secondary site, epithelial cells undergo a mesenchymal–epithelial transition (MET), and the mesenchymal state is reverted to a more epithelial-like state, facilitating metastatic colonization [35]. However, not all CTCs possess the capacity to induce metastasis. In some patients, CTCs are present in the absence of detectable metastasis [38].

It is feasible to differentiate between mesenchymal, epithelial, and hybrid E/M CTC phenotypes that have both EM and ME potential [38]. Different EMT phenotypes and dynamics can confer different metastatic abilities to tumor cells. The hybrid phenotype is more aggressive and more plastic, with stem cell-like properties, while the full M phenotype exhibits high expression of mesenchymal cell markers. This type is highly invasive but exhibits reduced metastatic potential due to a restricted cellular plasticity that impedes the reactivation of the proliferative state [35]. One linear tracing study has shown that EMT occurs at a clonal level and that the intraclonal EMT frequency in different clone populations in BC contributes to cancer progression [41]. However, the important question is how different cancer cells display distinct EMT types within the TMA. Understanding the cell-intrinsic and microenvironment regulation of these states is of particular significance.

In addition, certain CSCs, after undergoing the EMT process, detach from the primary cancer site, enter the circulation and spread to a secondary site [38]. The ability of CSCs to transition between EMT and MET cell phenotypes determines their metastatic characteristics, enabling them to adapt to a new environment in the metastatic site [42]. The primary functions of CSCs are colonization and outgrowth at the metastatic site.

On balance, metastasis dissemination is a complex process driven by cellular plasticity and environmental factors, rather than just stemness-like traits. In contrast, CSC-like phenotypes are characterized by self-renewal and differentiation. These traits are essential for rapid proliferation, expansion, and colonization of secondary sites, leading to a higher metastatic burden and increased ALN count. This may explain why stemness markers are strongly correlated with a higher number of positive ALNs but may not serve as definitive markers for the initial onset of the metastatic spread itself.

Our study makes important contributions to the literature as, to the best of our knowledge, limited attention has been dedicated to the co-expression of CD44 and CD117 in BC. However, CD44 and CD117 co-expression has been associated with aggressive behavior in several solid tumors (ovarian, colorectal, and prostate cancer), providing a plausible biological basis for the present study. Of particular significance are the findings demonstrating that this co-expression is significantly associated with the number and percentage of involved ALNs, although it is not correlated with metastasis. When interpreted in the context of the EMT, this dichotomy provides a novel and compelling basis for further investigation into how these stemness markers are correlated with distinct EMT phenotypes in BC.

The main limitation of this study is the use of immunohistochemical assessment based on a single tissue slide, which may not reflect intra-tumoral heterogeneity. Additionally, the study sample was derived from a single center, and its size was relatively small. Consequently, larger multicenter studies incorporating additional stem cell markers and, above all, transcription factors are needed to confirm and further explore these observations.

## 5. Conclusions

CD44 and CD117 co-expression is independently associated with a higher number and percentage of metastatic ALNs, suggesting its potential role as a marker of tumor aggressiveness and its potential prognostic significance. However, further studies are required to validate these results and clarify the involved mechanisms in the context of tumor immunology and the overall tumor biology of different IDC subtypes.

## Figures and Tables

**Table 1:** Links between expression of CD44 and CD117 and axillary lymph node status.

Expression Level	ALN Status		Total	*p*
	Negative (n = 59)	Positive (n = 77)		
CD44				0.893
Low	42 (71.2%)	54 (70.1%)	96 (70.6%)	
High	17 (28.8%)	23 (29.9%)	40 (29.4%)	
CD117				0.504
Low	48 (81.4%)	59 (76.6%)	107 (78.7%)	
High	11 (18.6%)	18 (23.4%)	29 (21.3%)	
Number of highly expressed membrane markers				0.763
0	35 (59.3%)	44 (57.1%)	79 (58.1%)	
1	20 (33.9%)	25 (32.5%)	45 (33.1%)	
2	4 (6.8%)	8 (10.4%)	12 (8.8%)	

**Table 2:** Clinicopathological parameters related to CD44 expression, CD117 expression and co-expression of CD44 and CD117.

Tumor Characteristics	CD44		*p* *	CD117		*p* *	High Expression of Membrane Proteins		*p* *
	Low	High		Low	High		0 or 1 Marker	2 Markers	
Tumor size			0.651			0.832			0.824
T1	32 (33.3%)	12 (30.0%)		33 (30.8%)	11 (37.9%)		40 (32.3%)	4 (33.3%)	
T2	46 (47.9%)	22 (55.0%)		55 (51.4%)	13 (44.8%)		61 (49.2%)	7 (58.3%)	
T3	3 (3.1%)	0 (0.0%)		2 (1.9%)	1 (3.4%)		3 (2.4%)	0 (0.0%)	
T4	15 (15.6%)	6 (15.0%)		17 (15.9%)	4 (13.8%)		20 (16.1%)	1 (8.3%)	
ALN status			0.893			0.504			0.462
Negative	42 (43.8%)	17 (42.5%)		48 (44.9%)	11 (37.9%)		55 (44.4%)	4 (33.3%)	
Positive	54 (56.3%)	23 (57.5%)		59 (55.1%)	18 (62.1%)		69 (55.6%)	8 (66.7%)	
ALN pN stage			0.966			0.554			0.656
pN0	42 (43.8%)	17 (42.5%)		48 (44.9%)	11 (37.9%)		55 (44.4%)	4 (33.3%)	
pN1	44 (45.8%)	18 (45.0%)		48 (44.9%)	14 (48.3%)		57 (46.0%)	5 (41.7%)	
pN2.3	10 (10.4%)	5 (12.5%)		11 (10.3%)	4 (13.8%)		12 (9.7%)	3 (25.0%)	
Number of involved ALN **	1.84 ± 2.49 2.0 (0.0–10.0)	2.93 ± 4.89 2.0 (0.0–19.0)	0.090	1.88 ± 2.74 1.0 (0.0–16.0)	3.21 ± 5.07 2.0 (0.0–19.0)	0.061	1.85 ± 2.66 1.0(0.0–16.0)	5.33 ± 7.06 2.0 (0.19.0)	0.001
% of metastatic ALN **	14.37 ± 18.75 7.2 (0.0–100)	18.92 ± 29.49 7.5 (0.0–100)	0.283	14.35 ± 20.11 6.2 (0.0–100)	20.73 ± 29.34 8.7 (0.0–100)	0.175	14.20 ± 19.46 6.7 (0.0–100)	31.37 ± 40.54 11.5 (0.0–100)	0.011
Histological grade			0.571			0.094			0.052
G1	5 (5.2%)	2 (5.0%)		6 (5.6%)	1 (3.4%)		7 (5.6%)	0 (0.0%)	
G2	66 (68.8%)	24 (60.0%)		75 (70.1%)	15 (51.7%)		85 (68.5%)	5 (41.7%)	
G3	25 (26.0%)	14 (35.0%)		26 (24.3%)	13 (44.8%)		32 (25.8%)	7 (58.3%)	
Nottingham score **	6.90 ± 1.12 7.0 (3.0–9.0)	6.85 ± 1.15 7.0 (4.0–8.0)	0.829	6.79 ± 1.14 7.0 (3.0–9.0)	7.24 ± 0.99 7.0 (4.0–8.0)	0.052	6.84 ± 1.11 7.0 (3.0–8.0)	7.33 ± 1.16 8.0 (4.0–8.0)	0.146
ER score **	4.28 ± 3.76 6.5 (0.0–8.0)	2.68 ± 3.71 1.0 (0.0–8.0)	0.024	4.41 ± 3.75 7.0 (0.0–8.0)	1.59 ± 3.17 1.0 (0.0–8.0)	<0.001	4.12 ± 3.80 6.0 (0.0–8.0)	0.58 ± 2.02 1.0 (0.0–0.7)	0.002
PR score **	3.92 ± 3.52 5.5 (0.0–8.0)	2.28 ± 3.23 1.0 (0.0–8.0)	0.012	4.01 ± 3.50 6.0 (0.0–8.0)	1.31 ± 2.66 1.0 (0.0–8.0)	<0.001	3.73 ± 3.52 5.0 (0.0–8.0)	0.42 ± 1.44 0.0 (0.0–5.0)	0.002
Ki-67 index (%) **	26.53 ± 13.73 24.0 (10.0–68.0)	33.23 ± 17.84 29.5 (9.0–77.0)	0.020	26.15 ± 13.69 22.0 (9.0–77.0)	37.17 ± 17.90 35.0 (12.0–68.0)	<0.001	27.29 ± 14.52 25.0 (9.0–77.0)	41.00 ± 18.12 47.5 (12.0–68.0)	0.003
Molecular subtype			0.006			<0.001			<0.001
TNBC	18 (18.8%)	18 (45.0%)		17 (15.9%)	19 (65.5%)		26 (21.0%)	10 (83.3%)	
HER2+	23 (24.0%)	8 (20.0%)		27 (25.2%)	4 (13.8%)		30 (24.2%)	1 (8.3%)	
Luminal A	55 (57.3%)	14 (35.0%)		63 (58.9%)	6 (20.7%)		68 (54.8%)	1 (8.3%)	

* *p*-values shown are unadjusted. A Bonferroni correction was additionally performed as a sensitivity analysis. ** Continuous variables are presented as mean ± standard deviation and median (minimum–maximum). Comparisons were performed using the Mann–Whitney U test. Categorical variables are presented as number (percent). Comparisons were performed using chi-square or Fisher’s exact test, as appropriate. *p*-values for variables with more than two categories represent overall comparisons across all subgroups.

**Table 3:** Association of clinicopathological parameters, molecular subtype of cancer and expression of CD44 and CD117 with positive ALN status—results of logistic regression analysis.

Tumor Characteristics	OR	95% CI	*p*
*Univariate analysis*			
Tumor size			
T1	reference *		
T2	1.614	0.752–3.463	0.219
T3	0.001	0.000–10.033	0.999
T4	7.200	1.850–28.016	0.004
Histological grade			
G1	reference *		
G2	0.643	0.127–3.261	0.594
G3	1.228	0.576–2.617	0.595
Nottingham score	0.866	0.637–1.178	0.360
ER score	0.985	0.901–1.077	0.740
PR score	0.977	0.887–1.077	0.642
Ki-67 index (%)	1.022	1.001–1.044	0.049
Molecular subtype			
TNBC	reference *		
HER2+	0.989	0.374–2.618	0.982
Luminal A	0.876	0.388–1.978	0.749
High CD44 expression	1.052	0.499–2.217	0.893
High CD117 expression	1.331	0.574–3.088	0.505
Number of highly expressed membrane markers	1.147	0.679–1.935	0.609
Coexpression of membrane markers	1.594	0.456–5.572	0.465
*Multivariate analysis*			
T4 tumor size	7.200	1.850–28.016	0.004

* Reference categories: T1 tumor size, G1 histological grade, TNBC molecular subtype. All other categories were compared against the reference categories.

**Table 4:** Association of clinicopathological parameters, molecular subtype of cancer, and expression of CD44 and CD117 with number of involved ALNs—results of linear regression analysis.

Tumor Characteristics	B	95% CI	*p*
*Univariate analysis*			
Tumor size			
T1	reference *		
T2	0.029	−1.125–1.184	0.960
T3	−2.211	−6.123–1.702	0.266
T4	1.498	−0.079–3.075	0.062
Histological grade			
G1	reference *		
G2	0.113	−1.107–1.333	0.855
G3	0.169	−1.107–1.445	0.794
Nottingham score	0.045	−0.471–0.561	0.865
ER score	−0.151	−0.302−0.001	0.048
PR score	−0.189	−0.351−0.027	0.022
Ki-67 index (%)	0.016	−0.022–0.054	0.396
Molecular subtype			
TNBC	reference *		
HER2+	0.334	−1.041–1.709	0.632
Luminal A	−1.093	−2.233–0.046	0.060
High CD44 expression	1.081	−0.172–2.335	0.090
High CD117 expression	1.328	−0.063–2.719	0.061
Number of highly expressed membrane markers	1.049	0.183–1.915	0.018
Co-expression of membrane markers	3.478	1.532–5.425	0.001
*Multivariate analysis*			
T4 tumor size	1.671	0.162–3.181	0.030
Co-expression of membrane markers	3.609	1.686–5.532	<0.001

* Reference categories: T1 tumor size, G1 histological grade, TNBC molecular subtype. All other categories were compared against the reference categories.

**Table 5:** Negative binomial regression analysis for number of involved ALNs.

Tumor Characteristics	IRR	95% CI	*p*
Tumor size			
T1	reference		
T2	1.570	0.943–2.618	0.083
T3 and T4	2.770	1.437–5.348	0.002
Histological grade			
G1	reference		
G2	1.524	0.490–4.739	0.467
G3	0.862	0.243–3.058	0.819
Ki-67 index (%)	1.030	1.007–1.054	0.011
Molecular subtype			
TNBC	reference		
HER2+	0.829	0.433–1.590	0.573
LuminalA	0.279	0.117–0.666	0.004
Co-expression of membrane markers	3.289	1.567–6.897	0.002

**Table 6:** Association of clinicopathological parameters, molecular cancer subtype, and expression of CD44 and CD117 with percentage of involved ALNs—results of linear regression analysis.

Tumor Characteristics	B	95% IC	*p*
*Univariate analysis*			
Tumor size			
T1	reference *		
T2	−2.703	−10.324–4.918	0.484
T3	−16.065	−41.912–9.782	0.221
T4	15.923	5.715–26.132	0.002
Histological grade			
G1	reference *		
G2	−0.146	−8.215–7.923	0.972
G3	1.996	−6.438–10.431	0.640
Nottingham score	1.096	−2.313–4.504	0.526
ER score	−1.066	−2.057–−0.075	0.035
PR score	−1.257	−2.327–−0.187	0.022
Ki-67 index (%)	0.130	−0.119–0.380	0.304
Molecular subtype			
TNBC	reference *		
HER2+	2.213	−6.880–11.305	0.631
Luminal A	−7.710	−15.231–−0.189	0.045
High CD44 expression	4.549	−3.793–12.891	0.283
High CD117 expression	6.386	−2.871–15.642	0.175
Number of highly expressed membrane markers	4.727	−1.063–10.517	0.109
Co-expression of membrane markers	17.170	4.034–30.306	0.011
*Multivariate analysis*			
T4	16.811	6.850–26.772	0.001
Coexpression of membrane markers	18.481	5.791–31.170	0.005

* Reference categories: T1 tumor size, G1 histological grade, TNBC molecular subtype. All other categories were compared against the reference categories.

**Table 7:** Negative binomial regression analysis with log-offset for the rate of metastatic ALNs relative to the total number of examined ALNs.

Tumor Characteristics	IRR	95% CI	*p*
Tumor size			
T1	reference		
T2	0.754	0.527–1.079	0.122
T3 and T4	1.305	0.832–2.049	0.247
Histological grade			
G1	reference		
G2	0.911	0.387–2.141	0.830
G3	0.693	0.268–1.789	0.449
Ki-67 index (%)	1.040	1.022–1.058	<0.001
Molecular subtype			
TNBC	reference		
HER2+	1.416	0.912–2.203	0.122
LuminalA	1.464	0.778–2.755	0.238
Co-expression of membrane markers	2.457	1.629–3.717	<0.001

## Data Availability

The data presented in this study are available from the corresponding author upon reasonable request.

## Abbreviations

The following abbreviations are used in this manuscript:

BC	breast cancer
CSCs	cancer stem cells
BCSCs	breast cancer stem cells
HA	hyaluronic acid
SCF	stem cell factor
IDC	invasive ductal cancer
TNBC	triple-negative breast cancer
ALN	axillary lymph node
EMT	epithelial–mesenchymal transition
MET	mesenchymal–epithelial transition
TMA	tumor microenvironment
CTCs	circulating tumor cells
